# Sampling Twitter users for social science research: evidence from a systematic review of the literature

**DOI:** 10.1007/s11135-023-01615-w

**Published:** 2023-01-27

**Authors:** Paula Vicente

**Affiliations:** Business Research Unit (bru_ISCTE), ISCTE-Instituto Universitário de Lisboa, Av. Forças Armadas, 1649-026 Lisboa, Portugal

**Keywords:** Sampling plan, Social science research, Twitter, User-level sampling

## Abstract

All social media platforms can be used to conduct social science research, but Twitter is the most popular as it provides its data via several Application Programming Interfaces, which allows qualitative and quantitative research to be conducted with its members. As Twitter is a huge universe, both in number of users and amount of data, sampling is generally required when using it for research purposes. Researchers only recently began to question whether tweet-level sampling—in which the tweet is the sampling unit—should be replaced by user-level sampling—in which the user is the sampling unit. The major rationale for this shift is that tweet-level sampling does not consider the fact that some core discussants on Twitter are much more active tweeters than other less active users, thus causing a sample biased towards the more active users. The knowledge on how to select representative samples of users in the Twitterverse is still insufficient despite its relevance for reliable and valid research outcomes. This paper contributes to this topic by presenting a systematic quantitative literature review of sampling plans designed and executed in the context of social science research in Twitter, including: (1) the definition of the target populations, (2) the sampling frames used to support sample selection, (3) the sampling methods used to obtain samples of Twitter users, (4) how data is collected from Twitter users, (5) the size of the samples, and (6) how research validity is addressed. This review can be a methodological guide for professionals and academics who want to conduct social science research involving Twitter users and the Twitterverse.

## Introduction

Social science research is the activity of gathering, analyzing, and interpreting information to understand the why, when, where, what and how of social relationships between humans and their interactions within society. Surveys are a key method when conducting cross-sectional quantitative research in the social science field and permit the study of populations that are too large to observe exhaustively and that have individual people as the unit of analysis (Babbie 2017, p. 270). Surveys involve the administration of standardized questionnaires to a sample of respondents for the systematic collection of a wide variety of unobservable data. It is extremely important to select a truly representative sample from the population of interest so that the inferences derived from the sample can be generalized back to the population.

Nowadays, surveys are up against the significant challenges of declining response rates and increased measurement errors, both with consequences on the validity of study results. The low response rate is due in part to the response burden; more specifically, surveys are reportedly boring, redundant, and frustrating for the respondents, and questionnaires are too long with questions people do not want to answer. Regarding measurement error, there is increasing concern about the quality of data because many respondents tend to avoid negative opinions and try to portray themselves in a socially desirable manner, which impedes the researchers from getting truthful responses and prejudices the validity of the outcomes of the survey research. Additionally, responses to survey questions often depend on subjects’ motivation, memory, and ability to respond. Particularly when dealing with events that happened in the distant past, respondents may not have a clear recollection of their own motivations or behaviors or perhaps their memory of the events has evolved with time and is no longer retrievable (Bhattacherjee 2012, p. 90; Couper 2013; Salganik 2018, p. 86). These weaknesses make it pertinent to try out novel approaches to social science research.

Social media provide exciting opportunities that can ‘‘open up a new era’’ of social science research (Salganik 2018, p. 2). The ability to aggregate vast amounts of digital traces of human behavior through social media platforms represents a new data collection paradigm for social science research (Salganik 2018, p. 13). Powerful computational resources combined with the availability of massive datasets have given rise to a growing body of work that uses a combination of machine learning, natural language processing, network analysis, and statistics for the measurement of population structure and human behavior on an unprecedented scale (Gilbert 2010; Stieglitz et al. 2014).

Twitter is one of the social media platforms that social scientists rely on to conduct research. With more than 400 million active monthly users that post 500 million tweets per day (Statista 2022c), Twitter is a huge database—both in number of users and amount of data—for conducting large-scale studies of human behavior. Twitter allows access to its data via several Application Programming Interfaces (APIs). Whereas academic researchers generally rely on one of these freely available data sources, social analytics industries and government entities buy in to get elevated access, e.g., to 10% of the overall Twitter data, also known as the Decahose API (Twitter 2022c). However, analyzing Twitter data to describe human behavior is complicated by several challenging factors, related above all to the representation of human populations and human behavior, as well as by methodological issues, such as coverage (e.g., Gayo-Avello 2011), measurement (e.g., Cohen and Ruths 2013; Lazer et al. 2014) and generalizability (e.g., Tufekci 2014).

Almost all of the analyses relying on Twitter depend on access to samples retrieved by resorting to a free or costly API. As Twitter does not reveal details about how sampling is handled by their APIs, the use of Twitter data is regarded as highly problematic from a data quality point of view, especially in the social sciences (Pfeffer et al. 2018). The prevalence of sampling means that researchers need, on one hand, to understand platform mechanisms and possible biases in the resulting data, and on the other hand, to question research validity—Is the data retrieved representative and reliable for phenomenon analysis?. There is evidence that the sampling strategy affects not only the representativeness of the data collected but also the substantive conclusions of analyses (Rafail 2018). Moreover, it is only recently that researchers have begun to question whether tweet-level sampling—in which the tweet is the sampling unit—should be replaced by user-level sampling—in which the sampling unit is the user (Zhang et al. 2018). The major rationale for this shift is that tweet-level sampling does not consider the fact that some core discussants on Twitter are much more active tweeters than other less active users, which means that discussion trends are dominated by these active users. On the other hand, user-level sampling can incorporate users’ sociodemographic characteristics as well as their engagement level on social media, thus providing more accurate insights into the phenomena under study (Zhang et al. 2018).

Though scarce, sound knowledge on how to design and implement a user-based sampling plan to obtain a sample of Twitter users with the desirable properties of representation and replication is of indisputable relevance, as reliable and valid information can only be achieved when based on methodologically sound research. Berzofsky et al. (2018) conducted research on the efficiency of probability sampling, but the focus was on a narrow population (persons aged 14–21). Rafail (2018) discusses the impact of non-random sampling strategies for different types of Twitter populations, and Hino and Fahey (2019) propose a strategy to archive a representative database of Twitter data by sampling Twitter accounts. However, a sampling plan is more than just the choice of a sampling method. It comprises a set of interrelated stages that includes population definition, constructing the sampling frame, decision about sample size, and choice of a data collection strategy.

The present work seeks to contribute to this topic by conducting a systematic literature review of sampling plans designed and executed to obtain samples of Twitter users for social science research. The remainder of the paper is structured as follows: Sect. 2 provides a literature review and a summary of related work; Sect. 3 describes the methodology used for the systematic literature review; Sect. 4 presents the results obtained; finally, Sect. 5 discusses the results and presents the conclusions.

## Background

### Surveys sampling plan

**Fig. 1:**
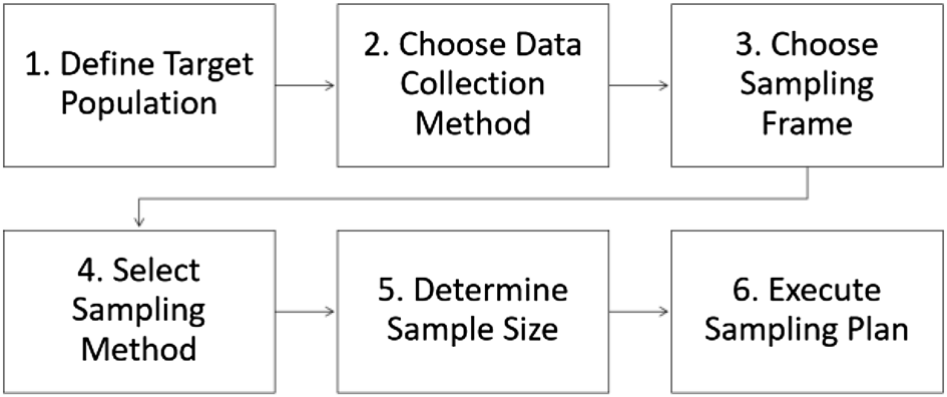
Sampling plan stages in surveys

The sampling plan is the process used to select a sample of units from the population for the purpose of making measurements and inferences about that population (Groves et al. 2009, p. 42). Figure 1 presents the typical sampling plan adopted in surveys. Despite the linear sequence suggested by the diagram, a change in the order of the stages or even the simultaneity of two stages is not uncommon since decisions are strongly interrelated (for example, the choice of the sampling frame and the sampling method, or the choice of the sampling method and the data collection method). A brief discussion of the issues in each stage is presented below.

Social science researchers interested in estimating the prevalence of a certain condition in the population would start by formally defining the target population, i.e., the set of statistical units about which information is to be sought.

Regarding data collection, the questionnaire is the key instrument used in surveys and it can be administered in person, via fixed or mobile phones or online. The adoption of multiple modes of data collection has become increasingly popular. Both concurrent and sequential multi-mode systems can be used, such as (De Leeuw 2005): (i) One sample, one time period, one questionnaire but different modes for different sample persons; (ii) One sample, one time period, but different modes for different parts of the questionnaire (for the same person); (iii) One sample, multiple time points but the same persons measured with different modes at different time points, and (iv) Different samples, different modes, sometimes even different times and questionnaires.

Another stage in the sampling plan is the target population enumeration, which can be accomplished with a list of names, numbers, or other representation of the target population. That population enumeration is called the sampling frame because it is the list of units from which the sample is drawn. A perfect sampling frame is one in which each statistical unit in the population is separately listed once, only once, and no other irrelevant or extraneous elements are listed. However, not all sampling frames are perfect, and it takes effort and attention to review potential sampling frames to ensure that they are free from error or that the errors in the frames can be addressed. The sampling frame is ideally a full census of the target population but, in practice, it is typically a subset. When a material list does not exist, a conceptual list can be created by randomly generating numbers representing the units of the target population. Random Digit Dialling is an example of a conceptual sampling frame, built by randomly generating phone numbers, frequently used in telephone surveys (Waksberg 1978).

Sampling methods for drawing samples in the context of social sciences can be separated into random (emphasizing the representative relationship between the entire population and the selected sample), and non-random. In the first group, sampling eliminates the element of personal choice in selection and therefore removes subjective selection bias. Random sampling strengthens the belief that the sample is representative of the target population because there is an absence of selection bias. Non-random methods are a set of techniques where the researcher has some element of choice in the process. As inclusion is determined by a subjective criterion, it can be more difficult to extrapolate whether the sample accurately represents the larger population than when random sampling is used. The following are examples of non-random methods. Convenience sampling is an approach where units are selected based on the ease of access to the available group. Purposive sampling is an approach where units conform to certain predefined criteria for selection. In snowball sampling, the researchers usually start with a small number of initial contacts (seeds) who fit the research criteria and are invited to become participants within the research. The willing participants are then asked to recommend other contacts who fit the research criteria and who might also agree to be participants, who then in turn recommend other potential participants, and so on. Sampling usually finishes once either a target sample size or saturation point has been reached. Quota sampling is used to improve the representativeness of a sample to ensure that the relevant characteristics are present in sufficient quantities. If the sample has the same distribution as the target population, then it is likely to be representative of the target population (Babbie 2017, p. 198).

Determining the sample size entails considering several factors, namely: (i) the type of analysis being conducted—there are statistical procedures (e.g., regression analysis) that require a certain number of observations per variable—, (ii) population diversity—if the target population exhibits large variability in the behaviors and attitudes being researched, a larger sample is needed—, (iii) the tolerance for risk and the level of desired precision in the project—the more precision and the less risk of error we are willing to take, the larger the sample size must be—, and (iv) the available budget.

Finally, the execution of the sampling plan comprises implementing what was planned and designed in previous stages. In the end, we have a sample of respondents from which the data needed for the research project is, hopefully, successfully obtained.

### Populations in Twitter data

Three distinct statistical units are observed in Twitter data and they correspond to three distinct, but interrelated, populations (Eurostat 2018): the population of tweets, the population of accounts and the population of users. A tweet is a post on Twitter containing up to 280 characters that can include URLs and hashtags. The tweet may also include up to 4 photos, a GIF, or a video. Users must sign up to the platform to use it, and this can be done by creating a free account or buying an account. To keep an account active, the user must log on to the platform at least every 6 months (Twitter 2022d). One user can have several accounts (e.g., Joe Biden has his personal account—@JoeBiden—and an institutional account as President of the United States—@POTUS), and one account can have multiple users (e.g., business accounts may allow different staff members to access and post contents). Users may subscribe to other users' tweets, which is known as "following" and subscribers are known as "followers".

Twitter Inc. defines tweeting as a behavior “performed by people” as Twitter was created to be “a service for friends, family, and co-workers to communicate and stay connected through the exchange of quick, frequent messages.” (Twitter 2022e). However, the term “Twitter user” has expanded and today one can distinguish between two broad categories of users: (i) real-users, and (ii) digital-actors. Real-users represent human-beings, and digital-actors represent automated computer programs. Real-users can be described as (Uddin et al. 2014):

- *Personal users* casual home users who create their Twitter profile for fun, learning, or to acquire news, etc. These users do not strongly advocate any type of business or product, and their profiles are not affiliated with any organization. Generally, they have a personal profile and show a low to mild behavior in their social interaction.
- *Professional users* these are home users with professional intent on Twitter. They share useful information about specific topics and get involved in healthy discussion related to their area of interest and expertise. Professional users tend to be highly interactive; they follow many and are followed by many.
- *Business users* these users are different than personal/professional users in that they follow a marketing and business agenda on Twitter. The profile description strongly depicts their motive and a similar behavior can be observed in the way they tweet. Frequent tweeting and less interaction are two key factors that distinguish business users from both personal and professional users.

Digital actors are usually characterized by highly frequent tweeting, less or no interactivity, and their followers generally either increase (e.g., in case of feed/news users) or decrease (e.g., in case of spam users) over time. This type of user can be grouped into three different classes (Uddin et al. 2014):

- *Spam users* spammers mostly post malicious tweets at a fast rate, automated computer programs (bots) run behind a spam profile, and randomly follow users, expecting a few users to follow back. Sometimes, personal users can also behave as spammers, but they seldom get caught because their spamming behavior does not follow a pattern, which can be easily seen in the case of an automated spam profile. Moreover, followers of spam users decrease over time.
- *Feed/news* these profile types represent automated services that post tweets with information taken from news websites such as CNN, BBC, etc. or from different RSS (Really Simple Syndication) feeds. Like spammers, tweets posted by these profiles are often controlled by bots. The key difference between spammers and these profiles is the increase in the follower count over time. Moreover, these users are not interactive at all (i.e., zero replies).
- *Viral/marketing services* viral marketing, or advertising, refers to the marketing techniques that marketers use with the help of technologies/social networks to increase their brand awareness or sales, or to achieve other marketing objectives. People use a viral process, which is an advanced type of bot (i.e., an intelligent bot that spreads information and produces fake likes, followers, etc.), to accomplish their marketing tasks.

### Twitter data retrieval

Researchers’ interest in Twitter has been increasing partly because the platform makes its data easily available: any Twitter user can apply for a Developer Account which allows him/her to gain access to Twitter data using an API. Although Twitter itself offers APIs to allow the public to access their data, changes have been made to the terms of service to become more restrictive, especially since the Cambridge Analytica scandal (Bruns 2019). Whereas it was previously relatively straightforward to register as a Twitter developer and request one or more of the authentication keys required for Twitter apps that access the public Twitter API, the company now imposes a registration process requiring developers “to provide detailed information about how they use or intend to use Twitter’s APIs so that we can better ensure compliance with our policies. (…) Applications submitted with incomplete or insufficient information may be delayed while we request further information from a developer. Applications that do not comply with Twitter’s policies will be rejected.” (Roth and Johnson 2018).

Nevertheless, Twitter keeps two different families of APIs to allow the public to access their data: the Streaming API and the REST API. They differ in the functionality offered and the constraints on users (Bruns and Stieglitz 2013; Schwitter and Liebe 2020):

- *Streaming API* Twitter data can be accessed as a constant real-time data stream. It gives access to (a sample of) all tweets as they are published on Twitter. After sending a certain request to the API, specific data will be sent to the user continuously. The Streaming API only sends out real-time tweets. The Streaming API needs a search term to filter the results, such as a hashtag, a specific user ID or a geographical area defined by coordinates. Public statuses that match one or more of the filter predicates are returned.
- *REST API* retrieves data through the Search API (searching for tweets containing certain words, using specific hashtags, etc.) and the User API (collecting a user’s tweets, followers, etc.). In contrast to the Streaming API, which collects real-time data in an ongoing fashion, REST APIs are suitable for single searches of historical data. The most common cases include searching historic tweets and reading user profile information. The Search API retrieves past tweets that match the criteria within the search window available for Twitter searches (which covers a period ranging from a few days to several weeks depending on the frequency with which the search term occurred in recent tweets). Furthermore, rate limits must be considered. This API returns a maximum of 100 tweets per request with a limit of 180 requests in 15 min. Regarding user profile information, Twitter allows the collection of up to 3200 of the most recent tweets from a user’s individual timeline if the developer account accessing the API has access to that user (this either means the user must have a public profile or that the developer follows that user; in both cases, the developer cannot have been blocked by the user).

Although any registered researcher can access Twitter data for free via Twitter’s public API, there are companies licensed to access Twitter data who sell databases containing tweets and metadata at the request of researchers. Additionally, there are premium Twitter APIs to access historical archives of tweets that are only available on payment. The pricing however is prohibitive for most research and academic institutions (Bruns 2019).

All Twitter APIs that return tweets provide data and metadata. Data and Metadata are both forms of data, but they have different uses and different specifications. Data is simply the content that can provide a description, measurement, or even a report on anything relative to a person, event, or topic. Metadata is data about data. It means it is the description and context of the data which helps to organize, find, and understand data. Therefore, in addition to the text content itself, a tweet can have over 150 attributes associated with it such as (Twitter 2022b):

- “*Tweet Object*” the tweet object includes fundamental attributes such as *id, created_at,* and *text*. Tweet objects are also the ‘parent’ object to several child objects. Tweet child objects include *user*, *entities*, and *extended_entities*. Tweets that are geo-tagged will have a *place* child object.
- “*User Object*” the user object contains Twitter user account metadata that describes the Twitter user referenced. Users can author tweets, retweet, quote other users tweets, reply to tweets, follow users, be @mentioned in tweets and can be grouped into lists. In general, these *user* metadata values are relatively constant. Some fields never change, such as the user's *id* and when the account was created—*created_at.* Other metadata can occasionally change, such as the *screen_name,* display *name, description, location*, and other profile details. Some metadata frequently change, such as the number of tweets the account has posted *statuses_count* and its number of followers *followers_count*.
- “*Geo Object*” Tweets can be associated with a location, generating a tweet that has been ‘geo-tagged.’ Tweet locations can be assigned by using the Twitter user-interface or when posting a tweet using the API. Tweet locations can be an exact ‘point’ location—*coordinates*—or a Twitter place with a ‘bounding box’ that describes a larger area ranging from a venue to an entire region—*place*.

For years, users who chose to geotag tweets with any location, even somewhere as geographically broad as “New York City”, also automatically gave their precise GPS coordinates. Neither the users nor their followers would see the coordinates displayed on Twitter but the GPS information would still be included in the tweet’s metadata and was accessible through Twitter’s API. Twitter did not change this policy across its apps until April 2015. Users must now opt-in to share their precise location, but the GPS data people shared before the update still remains available through the API (Lapowsky 2019; Twitter 2022f).

### Sampling the Twitterverse: a parallel with surveys

While many features of online data collection are similar to surveys, a number of unique properties and new features account for methodological differences. Surveys produce self-report data, i.e., data that comes from asking respondents to recall wide-ranging aspects such as personality traits, moods, thoughts, attitudes, preferences, and behaviors, retrospectively with limited scope. In fact, much of social science knowledge and theory is based largely on self-report data. Social media provide the opportunity to record personal expression and human interaction in real time and on a large scale, without people actively doing anything. When using surveys, researchers have comparatively few respondents but a great deal of control over what information respondents provide. Although respondents provide information of interest to the researchers under these conditions, the limited sample size may not produce enough variability to study less commonly observed phenomena in their entirety. Social media data, in many ways, are precisely the opposite. They are completely unsolicited but offer unprecedented volume and variability (McCormick et al. 2017). In addition, social media data is quite often unable to answer specific questions posed by researchers and data users. This is because social media data, unlike survey data, are “found data” not “designed data.” (Couper 2013).

Despite their differences, both survey research and social media research face the challenge of data quality. Within the survey research tradition, the Total Survey Error (TSE) framework is the guidance for data quality taken by all researchers when designing and implementing the two dimensions of a survey: (a) the representation dimension, comprising all stages of the sampling plan, and (b) the measurement dimension, comprising all stages of the data measurement and collection process (Biemer 2010). As social media does not yet have a similar framework devoted to enumerating the error sources and the error generating processes, the data quality approach shaped by the TSE framework can be leveraged for this context (Biemer 2014; Callegaro and Yang 2018; Amaya et al. 2020). The sampling plan in Fig. 1 can be transposed to the Twitterverse, thus helping researchers to design and implement the process leading to a sample of Twitter users and the corresponding dataset. However, some adaptations must be made to address the main issues in a sampling plan, i.e., target population, sampling frame, sampling method, sample size and data collection design. Finally, a word must be said about research validity.

#### Target population

**Fig. 2:**
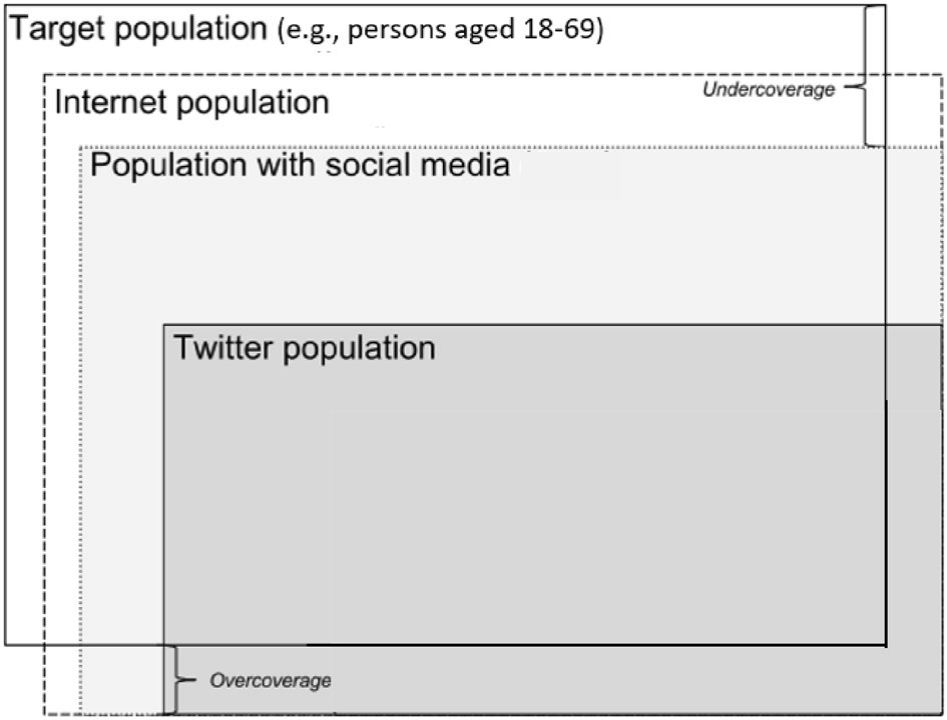
From target to Twitter population (adapted from Eurostat 2018)

Figure 2 shows the relationship between the target population of a social science project (e.g., persons aged 18–69 years) and the population observed on Twitter.

One can distinguish between the internet population—those with access to the internet—, the population with social media, and the Twitter population. The social media population suffers from under-coverage since not all people in the target population are social media users, and the under-coverage is even greater for the Twitter population since not all social media users are Twitter users. Likewise, over-coverage can occur to the extent that businesses and organizations maintain Twitter accounts (e.g., @mydeltaq or @FCGulbenkian). When the focus of the research is on individuals, businesses are not eligible and are a cause of over-coverage. Moreover, there are the digital-actors that do not correspond to human-beings and are for that reason out of the scope of studies targeted at populations of persons. Varol et al. (2017) estimate that the bot population on Twitter may range between 9 and 15%.

#### Sampling frames

In a study targeting the population of Twitter users, the ideal sampling frame would be a list of Twitter users—exhaustive, updated, and informative—from which a sample of tweeters is selected. However, Twitter, like other electronic populations, lacks a public membership list or a central registry like a phone directory (Andrews et al. 2003; Berzofsky et al. 2008; Couper and Miller 2008) and such a list is not made available by the platform either. As this makes it impossible to know the full population of Twitter users, creating a sampling frame to represent the general population of Twitter users is problematic. In such circumstances, researchers may resort to proxy populations for which a sampling frame is available, based on the presence or absence of critical attributes in the target population that must be mimicked (Lu and Franklin 2018), and from which a sample of Twitter users can be approached (e.g. a representative panel of the general adult population from which a sample of Twitter users is retrieved by imposing the condition “having an active Twitter account”).

#### Sampling methods

Random selection of a sample of Twitter users can only be accomplished when a sampling frame is available. If not, the selection must be non-random. However, in both cases there is the risk of selectivity bias caused by the decisions of the Twitter users that impact the likelihood of selection. As noted in Fig. 2, Twitter users are a subset of social media users, who are a subset of Internet users, who are in turn a subset of the whole target population. But it is not just a question of the percentage of users and non-users of internet, social media or Twitter: the frequency of usage and the level of proficiency in using these platforms are different across users and may have a non-ignorable effect on sample selection.

**Fig. 3:**
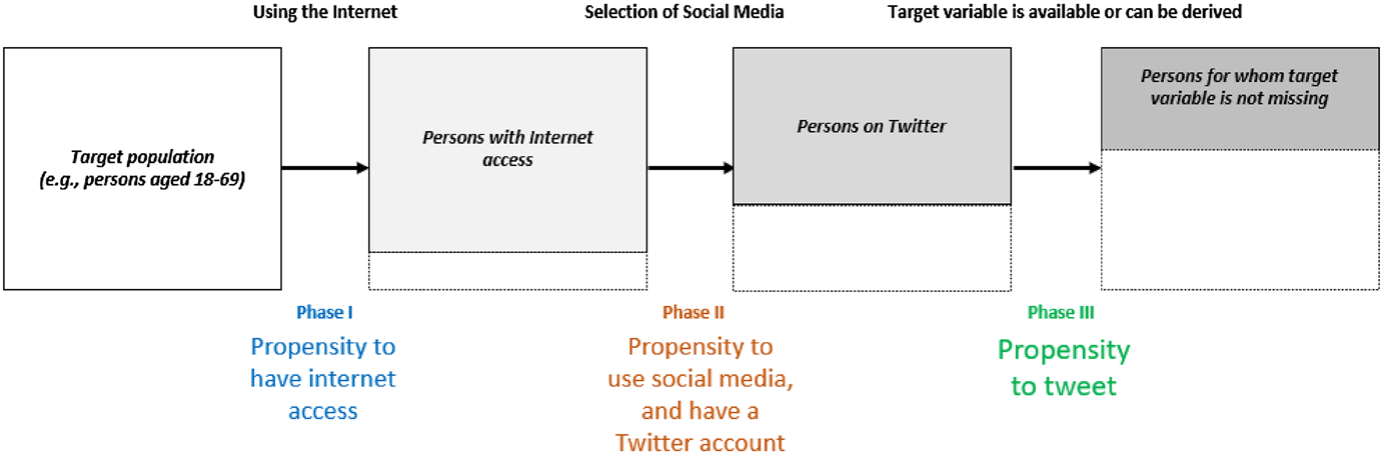
Self-selection mechanism in Twitter (Adapted from Eurostat 2018)

Figure 3 identifies three phases in the self-selection process present in Twitter data. Phase I and phase II are connected to the coverage error, i.e., the extent to which the frame population adequately covers the target population. As of January 2021, only 59.5% of the global population used the internet (Statista 2022a). According to DataReportal (2022), well over 9 out of 10 internet users now use social media but only 7% of social media users are Twitter users (Statcounter 2022). In this scenario, this restricted sampling frame may not be problematic if the research conclusions and the questions that generated them are limited to the Twitter universe. However, limiting the sampling frame to the users of Twitter in a study in which the interest is in generalizing findings to the offline population may raise issues of validity.

The population represented by Twitter data is Twitter users, who are disproportionately younger adults—more than 80% of users are under the age of 50 (Hootsuite 2020), therefore, if inferences are to be made to the general population, older adults will be under-represented, and the parameter of interest will be over or underestimated, i.e., the outcomes will be biased (Amaya et al. 2020). Beside the age bias, Twitter’s audience tends to be more educated and wealthier than the rest of the population, at least in the UK and US (Blank 2017; Pew Research Center 2019). Additionally, the use of online social media, and Twitter, is dependent on the socioeconomic background of individuals and their skills to use, first, the Internet and, secondly, social media platforms (Hargittai 2020), which is likely to make the sample of users of social media and of Twitter unbalanced. Moreover, tweeters’ profiles are not equal across countries: Pew Research Center (2019, 2021a) found no significant gender difference in Twitter users in the US although the Twitter’s global audience skews male—62% male versus 38% female (Hootsuite 2020).

Phase III relates to any decision of individuals that affects their presence in the sample. Social media is self-selective in the sense that not everybody posts messages on social media platforms, and those who do, do so at varying rates, from an occasional message from time to time to many messages a day (Nielsen 2006). In the US, 53% of Twitter users access the platform only a few times a week or less, and a minority of extremely active tweeters produce almost all tweets: the top 25% of users (the top is measured by tweet volume) produces 97% of all tweets. It is also important to note that those 25% of users do not exclusively produce original tweets—80% of tweets from this group are either direct retweets (49%) or replies to other tweets (33%). Replies and retweets are also what less-active tweeters tend to do (Bruns and Stieglitz 2013; Pew Research Center 2021b). Moreover, there is evidence that the content generated by users who tweet very often and users who rarely tweet is significantly different, which should be a cause of concern when creating predictive models based on aggregated data (e.g., Mustafaraj et al. 2011). Individuals who never or rarely post information may be invisible to certain sampling techniques. Those users might be systematically under-sampled, and this process can bias the results of data collection toward the heaviest users. In addition, not everybody reveals the same kind of information in their profile—some biography profiles are information-rich, but others are not—which means that it is difficult to execute quota sampling or to assess sample bias when the value of a target variable and/or an auxiliary variable is missing (Daas et al. 2016; Eurostat 2018).

#### Sample size

The decision about sample size when sampling the Twitterverse raises less concern than in surveys. While cost and time constraints pose limits on the number of subjects in the sample in surveys, these kinds of limitation do not exist (or are not so prominent) in social media. Social media generates datasets with millions of users or millions of data points about thousands of users and the access to these resources is wide and easy in most cases (there may be exceptions due to the API system used, for instance). It might be thought that results can be generalized due to the sheer number of observations. However, this can be a mistake, depending on methodological details independent of sample size. Numbers are not the only factor to consider when establishing the generalizability of a study and bigger is not always better (Anderson 2008; Hargittai 2015).

#### Data collection

The data that is generally of interest to social science researchers when investigating the Twitterverse is posted in the platform, i.e., the text of the tweets, or images, or files, uploaded and shared, or even metadata associated with tweets or users. This data is accessed using API systems, but this poses significant problems with regard to access to representative, high-quality data for analysis. Cheap, publicly available data such as that obtained from Twitter's public APIs is often of low quality, while high-quality data is expensive both financially and computationally. The free Streaming API provides only real-time data, is limited to about 1% of Twitter traffic and does not provide a representative sample of tweets in many cases (Hino and Fahey 2019). In addition, Twitter is quite often unable to answer specific questions posed by researchers and data users—because it is not “designed data” (Couper 2013)—which makes resorting to other data sources mandatory to compensate for the weaknesses of the Twitter datasets (Callegaro and Yang 2018).

#### Research validity

Researchers must scrutinize the research undertaken on Twitter for a variety of possible methodological pitfalls. The design and execution of the sampling plan largely determine the validity of the research findings, above all the external validity. Checking external validity requires focus on the ways in which the findings may not represent the broader population or context (Olteanu et al. 2019), therefore social scientists must inquire: (a) to what extent does an artificial situation like Twitter correctly reflect a broader real-world phenomenon?; (b) to what extent can the effects observed on the Twitter platform manifest differently on other platforms due to different functionalities, communities, or cultural norms?; (c) to what extent do the chosen sampling method and sample size affect sample representativeness, and (d) to what extent may constructs change over time and invalidate previous conclusions about societal and/or platform phenomena? The last question is only relevant in longitudinal studies but the other three are mandatory in all research.

## Method

### Research questions

The main goal of this systematic literature review is to disclose the design and execution of sampling plans in the context of applied social science investigation on Twitter. For this purpose, the main research questions were as follow:

1. How is the target population defined?
2. Which sampling frames exist and are used to represent the target population?
3. Which sampling methods and sampling strategies are implemented to select Twitter users?
4. How is data collected from Twitter users?
5. What is the size of the samples?
6. How is research validity addressed?

Additionally, some further information was extracted from the studies to complement the results:

- The purpose of the studies (exploratory, descriptive, or explanatory)
- The domain of the studies (e.g., political science, sociology, …)
- The geographic spread of the studies

### Search strategy

A standard systematic quantitative literature review methodology (Pickering and Byrne 2014) was followed with the aim of identifying all the relevant articles published in peer-reviewed journals in the subject area of social science research involving samples of Twitter users. Books, theses, dissertations, editorials, and conference papers were excluded. The searches covered any part of the paper (title, abstract and full text).

#### Databases searched

Eight databases were searched, including some of the main databases for social sciences. Specifically, the search was performed in: ACM Digital Library, Oxford Academic, Sage, Science Direct, Scopus, Springer, Taylor and Francis, and Web of Science.

#### Search terms

To perform the search in the databases, the focus was set on two main terms of interest: “Twitter” and “sampling”. A preliminary search based solely on these two terms yielded more than 20 thousand articles in the set of eight databases. An analysis of the first twenty results retrieved in each database revealed that most of the publications were off the goal, and further filtration was needed to ensure that included articles serve the objectives of this survey. Therefore, the search terms were reformulated to be more specific. This was done by using the term “sample of Twitter users” and several alternative terms to define the search queries, namely:

- “sample of twitter users” OR “sample of tweeters"
- “sampling twitter users” OR “sampling tweeters”
- “selection of twitter users” OR “selection of tweeters”

Additionally, inclusion/exclusion criteria were employed, namely:

- *Inclusion criteria* (i) applied research papers containing evidence of Twitter users (i.e., individual persons) as the target population, and (ii) papers with the full text available (institutional access).
- *Exclusion criteria* (i) methodological papers, i.e., studies that despite resorting to sampling had the main purpose of investigating computational or engineering issues (e.g., network analysis, bot detection, deep learning applications) and (ii) literature reviews.

Although no time restrictions were set, the papers are necessarily posterior to 2006, the year when Twitter was launched.

### Data analysis

For each paper selected for the literature review, several pieces of information were recorded on a database. First, general information was extracted to contextualize each paper, namely paper title, journal title, publication year, author(s) affiliation (i.e., university, organization, …), geographical information of author(s), publisher, keywords. Then data was collected on each of the research questions. When available, any additional information providing a more in-depth review of the application of sampling strategies in the Twitterverse was collected. The data collected for the selected studies was classified according to the following criteria:

- The characteristics adopted to define the target population (addressing RQ1)
- The lists of Twitter users adopted to select samples (addressing RQ2)
- Sampling methods (random vs non-random) and sampling strategies (addressing RQ3)
- Modes of data collection and data collection strategies (addressing RQ4)
- The number of users selected (addressing RQ5)
- The discussion about the generalizability of the findings and the strategies to overcome limitations (addressing RQ6)
- The domain of the research, evaluated through corresponding author’s affiliation (university, faculty, department, research centre)
- The purpose of the research
- The country of the corresponding author

## Results

### Papers identified by the search

Papers were retrieved in March 2022 using the abovementioned search strategy. This first search identified 196 papers. After removing duplicates, the title and abstract were scanned: 20 papers were excluded either for not meeting the inclusion criteria (7 could not be fully accessed, 11 were not related to the Twitter platform) or for meeting the exclusion criteria (2 were literature reviews).

An additional full text review—with special focus on the Method sections—was performed to ensure the suitability of the papers. In this final review, 67 papers were excluded in line with the exclusion criteria: 39 methodological papers (not applied research); additionally, 23 studies did not meet inclusion criteria for having as the target unit organisations/companies or the tweets instead of the user (individual). Moreover, a decision was made on excluding 5 papers using experimental designs since sample selection in these cases is addressed differently from that of cross-sectional studies.

**Fig. 4:**
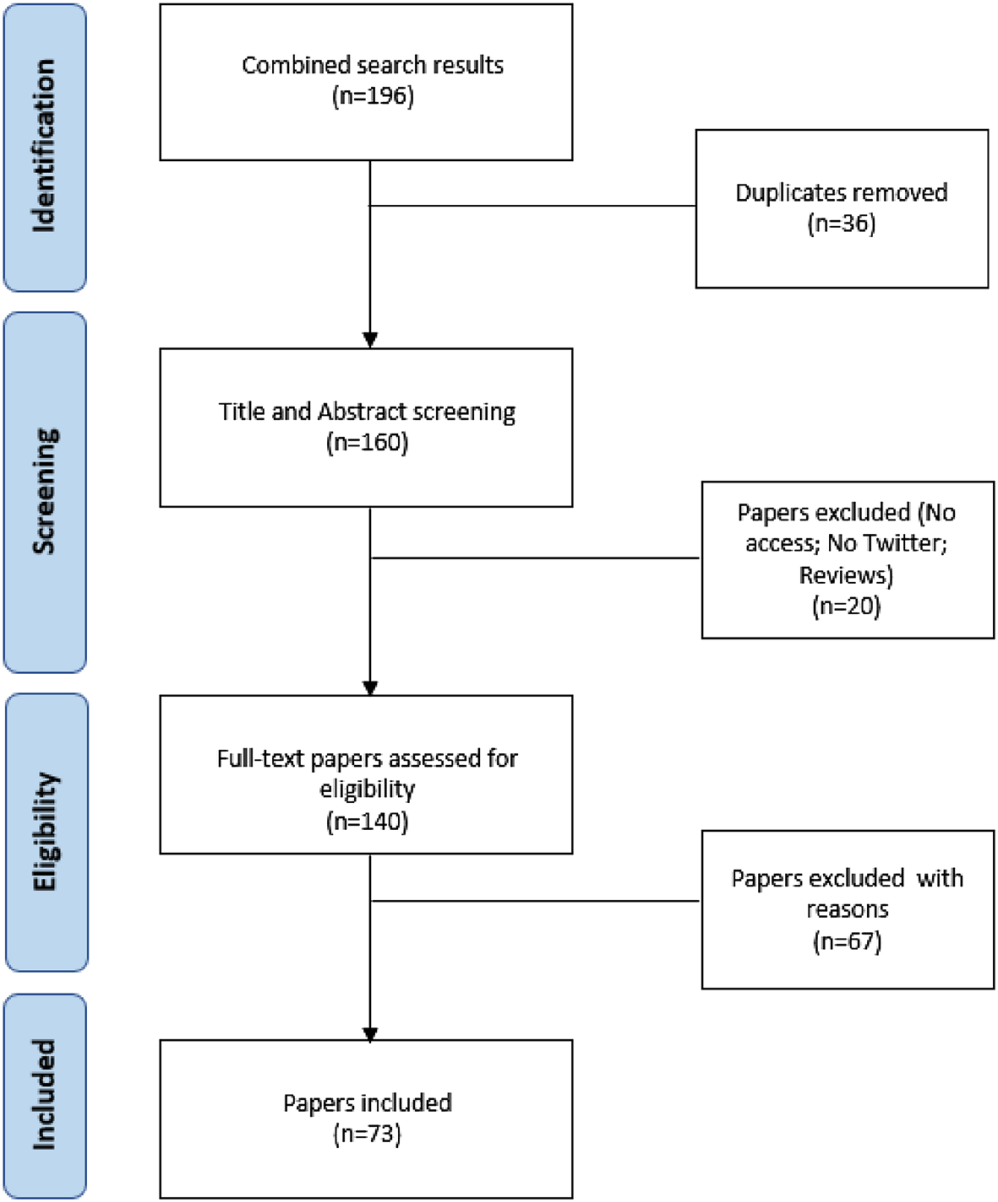
Flow chart of paper selection process for the systematic literature review

The final sample consists of 73 papers. Figure 4 summarizes the full selection process.

Table 1 shows the total number of papers identified in the search and that were retained for analysis from each database considered.

**Table 1:** Number of papers identified in search and retained from each database

Database	Number of papers identified in search	Number of papers retained
ACM	9	1
Oxford Academic	14	2
Sage	34	17
Science Direct	51	20
Scopus	26	5
Springer	14	3
Taylor and Francis	22	14
Web of Science	26	11
Total	196	73

### Papers’ spread

Most of the papers are published by corresponding authors from the United States (34), followed by United Kingdom (7), Hong Kong, Japan, and Spain (4 paper each), Germany and Israel (3 papers each), Belgium, and Chile (2 papers each) and with a single publication from Australia, Canada, China, Indonesia, Italy, Kingdom of Saudi Arabia, Singapore, South Korea, Switzerland and United Arab Emirates.

**Fig. 5:**
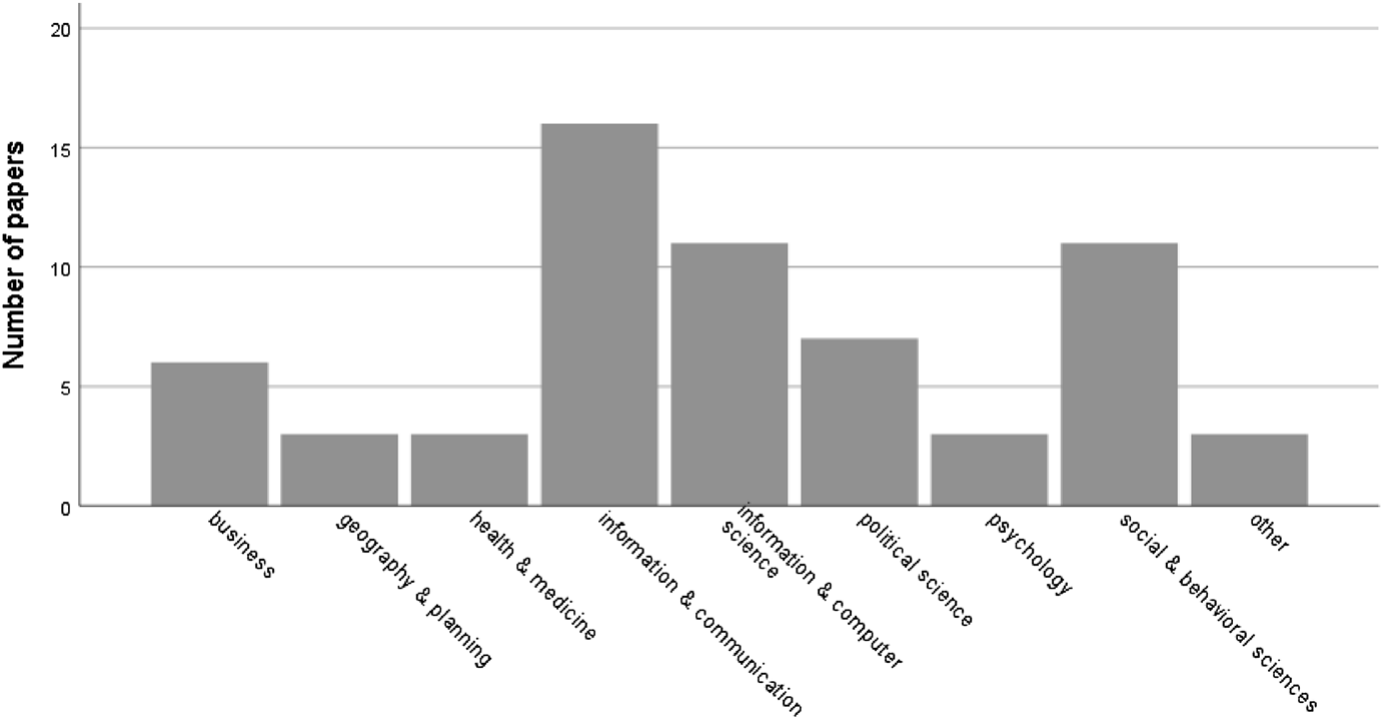
Scientific areas or domains of corresponding authors

Most of the corresponding authors (68) are affiliated to universities, and the areas or domains of the respective faculties, schools, or departments are mostly Information & Communication (16 studies), Information & Computer Science (11 studies), and Social & Behavioral Sciences (11 studies) (Fig. 5). It was not possible to identify the area/domain of the corresponding author in 10 papers due to a lack of information in the affiliation description.

**Fig. 6:**
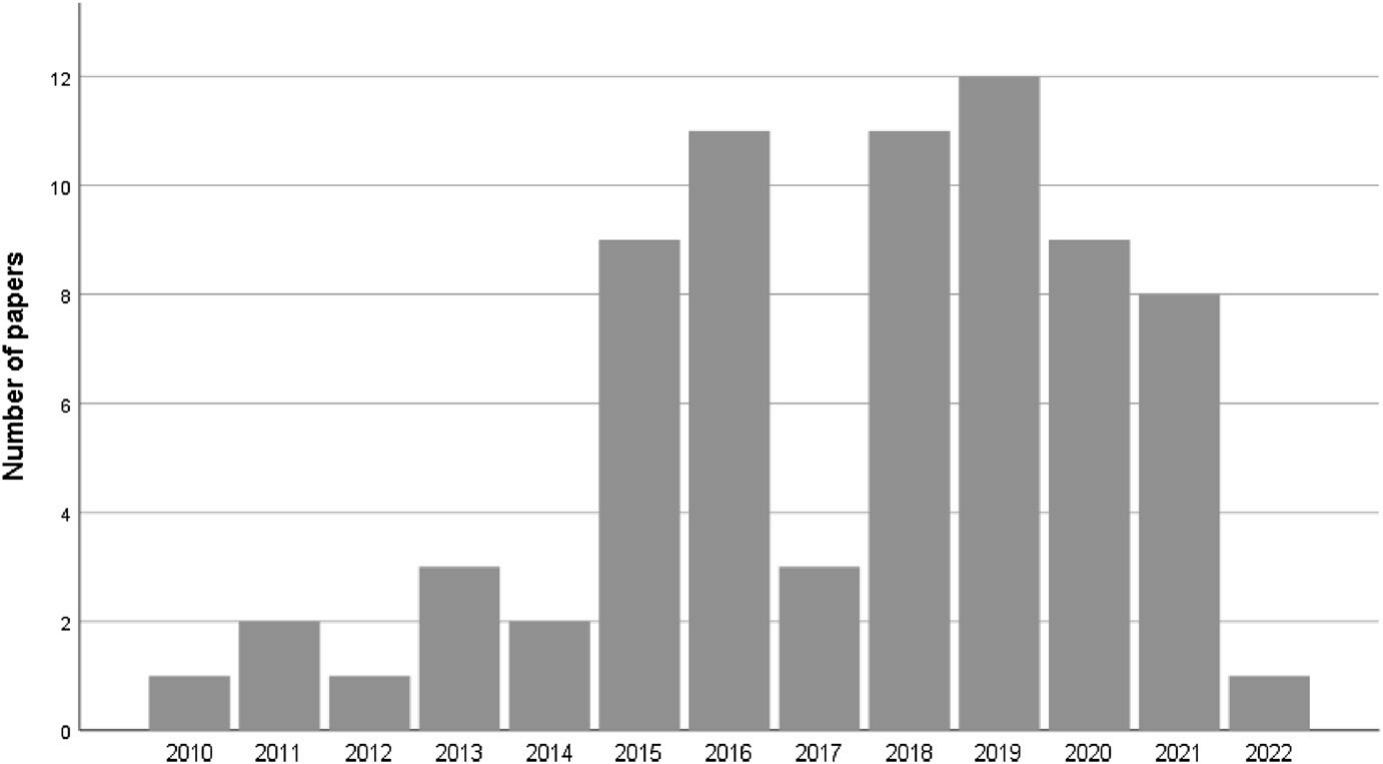
Number of selected papers per year of publication

Regarding the year of publication of the selected papers, Fig. 6 shows the number of papers selected for the literature review for each year of publication. The figure reveals an upward trend in the number of papers published involving applied research on the Twitterverse with samples of tweeters. Despite the low frequency in 2017 vis-a-vis the previous years, there is evidence of increased interest in Twitter as a resource among social scientists. The slight decrease in 2020–2021 may be the consequence of the COVID-19 pandemic which affected all activities. The 2022 bar covers only the 1st quarter of the year.

### Main purpose of the studies

The purpose of a study is a statement of the goal or why the study is being conducted. After considering all qualified papers, their purpose was mapped into 3 categories: exploratory, descriptive, and explanatory (Babbie 2017, p. 97):

- *Exploratory* information gathering aimed at discovering, uncovering, exploring the phenomena.
- *Descriptive* information gathering aimed at describing, summarizing the phenomena.
- *Explanatory* understanding and explaining the phenomena by looking at the relationships between them, and patterns among variables.

The two main research purposes found in the reviewed papers are explanatory (29 papers, 40%) and descriptive (28 papers, 38%). Table 2 presents two example studies, with quotations from the respective paper text, for each purpose category.

**Table 2:** Categories of research purposes for the selected papers and two example studies

Purpose category	Definition of purpose (examples)
Exploratory	Adopting an **exploratory approach**, we distinguish between health institutions, specialists, and advocates, and we assess key topics and framings promoted online by these actors. (Reveilhac and Lupton 2022) The current study was **exploratory** in nature with the aim to isolate a group of self-identified parents (i.e., stay-at-home parents) and examine their publicly available tweets concerning discipline and spanking. (Lee et al. 2020)
Descriptive	This study focuses on Twitter use during an eminent Belgian current affairs television (TV) programme to investigate **how** people talk about TV on Twitter. (D’Heer and Verdegem 2015) The current study was designed to **determine the use patterns and characteristics** of African American, Hispanic and White young adult Twitter users who reported past month blunt use. (Montgomery et al. 2018)
Explanatory	In this study, (…) we contribute to the extant literature (…) by **investigating the impact** of Twitter trust on both the intentions to continue using the platform and the intentions to follow and purchase other brands that are ‘‘hosted’’ on the Twitter platform. (Pentina et al. 2013) The aim of this paper is to approach concurrently these **various factors that influence** users' behaviour on social media during crises. (Mohammed and Ferraris 2021)

### Definition of the target population

This subsection responds to RQ1: How is the target population defined? Twitter users have the option to make their profile private; therefore, only the data of users without private profiles can be collected for research in any study involving the “population of Twitter users” with or without boundaries (Twitter 2022a).

Table 3 summarizes the features used to bound the target population and the number of studies that use each feature type and presents two examples for each.

**Table 3:** Feature types to delimit the population of Twitter users and two example studies

Feature types	Number of papers	Target population (examples)
No feature	18	This study’s primary interest is in **Twitter users**. (Fiesler and Proferes 2018) This study examined the ideological media consumption patters of **Twitter users.** (Shin 2020)
Tweet Object	18	Twitter users who **tweet about depression** (Cavazos-Rehg et al. 2016) Twitter users (…) who **posted tweets with #NBCFail** (O'Hallarn and Shapiro 2014)
User Object	17	Japanese Twitter users who **follow at least one media account and at least one member** of the Japanese Diet. (Kobayashi et al. 2019) Twitter users with **3 or more tweets** supporting ISIS (Torregrosa et al. 2020)
Geo Object	4	Twitter users who **tweeted from Los Angeles County** (Lerman et al. 2018) Twitter users whose **geo-tagged tweets were sent from the catchment area** (Pourebrahim et al. 2019)
Socio-demographic or other specific features	23	(…) Twitter users **with schizophrenia** (Hswen et al. 2017) **Israeli** Twitter users (Moshkovitz and Hayat 2021)

Eighteen of the reviewed studies define the target population simply as the general population of “Twitter users”. The remaining 55 studies use specific Twitterverse features to delimit the target population: tweet object features are found in 18 studies; user object features are found in 18 studies and geo object is the least used—only 4 studies delimit the population resorting to geographic criteria. Socio-demographic characteristics, such as age or country, or other specific characteristics (e.g., being a psychologist, being an asylum seeker) are used to bound the population in 23 studies. Age and region/country are the most frequently used (13 studies). Note that the definition of the target population involves more than one feature type in 7 studies; for example, in the case of Kobayashi et al. (2019) (Table 3) the population is bounded using a socio-demographic variable—country (“Japanese”)—and a user object feature—“follows at least one media account and at least one member of the Japanese Diet.”

### Sampling frames

**Fig. 7:**
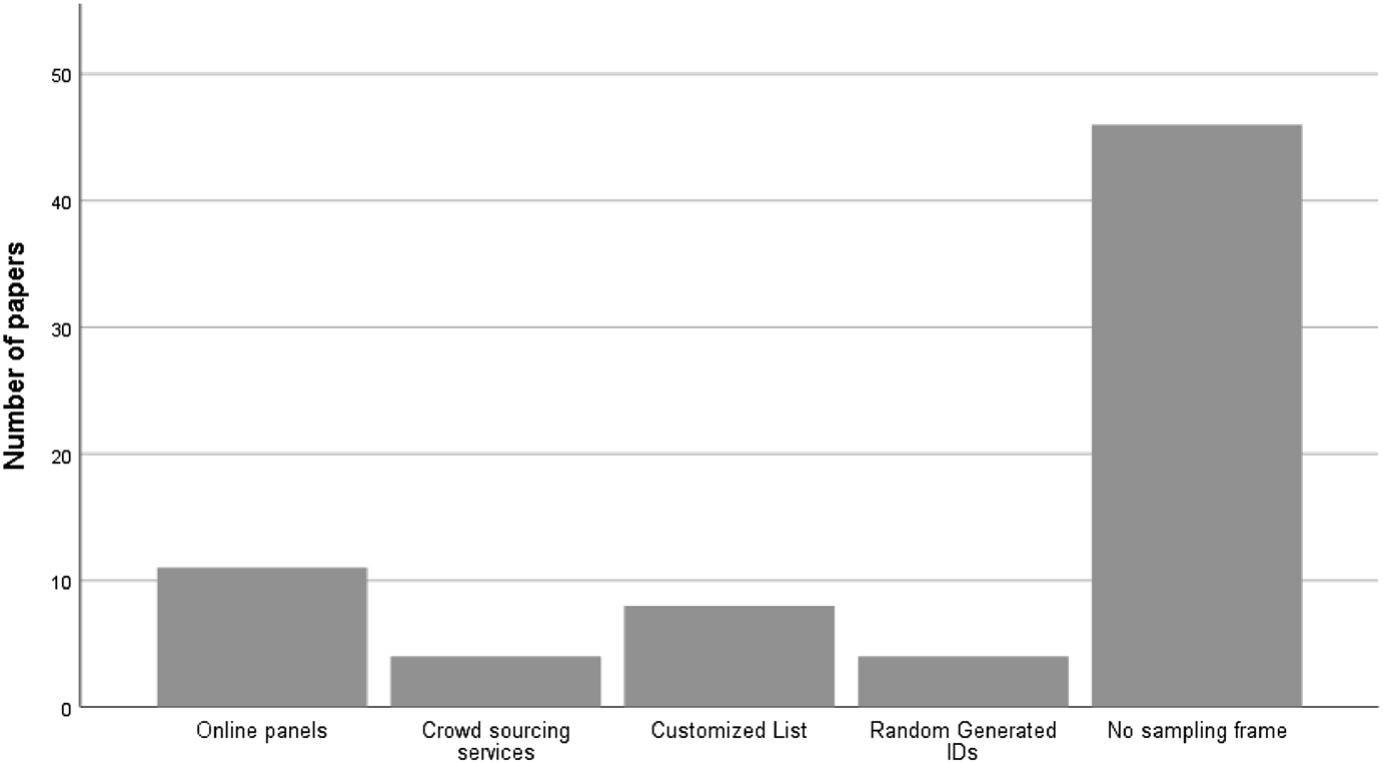
Sampling frames adopted in the selected papers

This subsection addresses RQ2: Which sampling frames exist and are used to represent the target population? Fig. 7 displays the distribution of the sampling frames adopted in the reviewed studies. The difficulty of building a sampling frame to list the Twitter users of the target population in an exhaustive and updated manner is evident. Most of the studies (46) were conducted without a sampling frame to support the sample selection of Twitter users.

In the remaining studies, which used a list to represent the target population, four different sampling frames can be distinguished:

- *Online panels* in 11 studies researchers resorted to research companies owning online panels (e.g., YouGov, Qualtrics), representative of the general adult population of a country, and requested a sample of Twitter users (e.g., Han et al. 2015; Sasaki et al. 2015).
- *Crowdsourcing services* services like Amazon Mechanical Turk or Yahoo! Crowd Sourcing Service are marketplaces for individuals and businesses to outsource their processes and jobs to a distributed workforce who can perform virtually any task. In 4 studies, researchers resorted to these services to request a sample of individuals interested in collaborating with the investigation (being a Twitter user was mandatory) (e.g., Tominaga et al. 2015; Fiesler 2018).
- *Customized list of Twitter users* in 8 studies the researchers built or acquired a list representing the target population of the study, such as a list of candidates to elections (Bouteca et al. 2017), a list of followers of 12 Twitter accounts (Kearney 2019) and a list of scientific tweeters (Yu et al. 2019).
- *Random Generated ID numbers* 4 studies adopted this kind of sampling frame, which consisted of creating a “list of Twitter users’ IDs” using a software system that randomly generates numbers, within (or not) a pre-specified range of numbers (e.g., Liang and Fu 2019). This process resembles Random Digit Dialling in telephone surveys (Waksberg 1978) and allows everyone with an active Twitter account to be represented, rather than only those who are in a list acquired to serve as a sampling frame. As in Random Digit Dialling, this approach is suitable for general population studies, which in the Twitterverse means targeting the “general population of Twitter users”.

Overall, most of the studies reviewed (63%) were conducted without a sampling frame and the remaining resorted to sampling frames that are external sources of the Twitterverse, either panels of research companies or recruitment from crowdsourcing platforms.

### Sampling methods and strategies

This subsection addresses RQ3: Which sampling methods and sampling strategies are implemented to select Twitter users?

The sampling methods in the reviewed papers can be mapped into two distinct categories: (i) methods supported by a sampling frame, and (ii) methods designed and implemented without a sampling frame. In the first category, when the sampling frame is a panel or a list of crowdsourcing members, sample selection is designed by setting conditions that sample members must meet, such as “being a Twitter user with a public profile”, “being a regular Twitter user”. These conditions are necessary since neither the panels nor the crowdsourcing platforms are made up solely of Twitter users. The researcher has no control over or intervention in the selection process. In studies with customized sampling frames, the sample of Twitter users is obtained by random selection (e.g., Bouteca et al. 2017) or by setting filters such as number (minimum or maximum) of followers or number of statuses posted (e.g., Kearney 2019). Finally, when the sampling frame comes from randomly generated numbers, the sample automatically includes those Twitter users whose ID numbers match the randomly generated numbers (e.g., Liang and Fu 2015; Liang et al. 2016).

When no sampling frame was available, the sample of users was selected either by non-random sampling methods or resorting to an API system search. The non-random sampling methods found in the reviewed papers were grouped in three main categories:

- *Convenience sampling* focuses on gaining information from Twitter users who are ‘convenient’ for the researcher to access. In the reviewed papers, convenience sampling was adopted in 6 studies, using one of the following approaches: (i) selection via researchers’ followers (e.g., Akyuz et al. 2021); (ii) selection via promoted or ad tweets (e.g., Montgomery et al. 2018); and (iii) selection of students on campus (e.g., Mohammed and Ferraris 2021).
- *Snowball sampling* this method is a kind of convenience sampling in which, after agreeing to cooperate, the Twitter users initially selected for the study are solicited to retweet the invitation to the study to their followers. In the revised studies, snowball sampling was adopted in 4 studies (e.g., Chen 2011; Pentina et al. 2013).
- *Purposive sampling* sample members are chosen based on the researcher’s sound judgment of what is a representative sample. Only 3 studies employed purposive sampling: in Sharples (2021), the researcher intentionally chose a sample of four Twitter handles based on the frequency of posts; in Jünger and Fähnrich (2020), sample members are the followers of a specific Twitter account and in Brady (2016), Twitter users are chosen based on specified profile features.

A significant number of studies (31) designed and implemented sample selection based on an initial search in Twitter data (Table 4). Keywords, hashtags, or geo-tags were used to query Twitter’s API and identify those Twitter users who posted messages related to the topic or event under study. The Twitter users were pinpointed based on topic-related keywords or topical hashtags contained in the messages they posted during a delimited period. This strategy is used mostly in studies intended to target an audience narrowed down to those who tweeted during a certain event or regarding a certain topic, such as smoking (Hswen et al. 2017), health conditions (Zhang and Ahmed 2019), ISIS support (Torregrosa et al. 2020), and natural disasters (Bica et al. 2021). Instead of hashtags or keywords, an API search can focus on tweets from specific locations (Murty et al. 2016, Osorio-Arjona and García-Palomares 2019) or followers of specific accounts (Hayat et al. 2016). The strategy of reaching a sample of Twitter users by simply searching data via an API system was adopted in 25 studies (Table 4).

**Table 4:** Sampling methods and sampling strategies in studies without a sampling frame

Method or strategy	Number of studies
Convenience sampling	6
Snowball sampling	4
Purposive sampling	3
Search via API system → Twitter users	25
Search via API system → Twitter users + Random sampling	4
Search via API system → Twitter users + Snowball sampling	2

Another sampling strategy based on API systems consisted of locating an initial sample of Twitter users via an API search and, in a subsequent stage, selecting a random subsample of those users because the initial set was found to be too large for the analysis in question (e.g., Vaccari et al. 2016; Baik et al. 2021). In another two studies, the opposite strategy was found: after locating an initial sample of Twitter users via an API system search, snowball sampling was adopted to increase sample size since the initial sample was found not to be large enough for the study objectives (O'Hallarn and Shapiro 2014; Lee et al. 2020).

Table 4 summarizes the sampling methods and the sampling strategies adopted in the reviewed studies when no sampling frame is available. Note that two of these studies did not have enough information to allow a full understanding of how the sample was selected.

### Data collection

This subsection provides answers to RQ4: How is data collected from Twitter users? Despite the richness of Twitter data, this data may not be enough for the study purposes, and it may be necessary to resort to other data sources to supplement and enrich the data gathered on the Twitterverse.

The reviewed papers include solely cross-sectional studies which means that data refer to “one time point”. It is important to note that it is necessary to specify the search’s time range when retrieving data and metadata from the Twitterverse. Although the time range of the studies varied from a few seconds to several months, the data is regarded as “one time point” as a trend evaluation of the phenomenon was never the study objective.

The data collection designs found in the reviewed studies are classified distinguishing between single-mode designs and mixed-mode designs. Three studies did not provide enough information to allow an understanding of how data collection was implemented.

#### Single mode, one sample

Under this design, data is collected for all sample members using a single mode of data collection. In the reviewed papers, this design was implemented using one of the following strategies:

- *Data collection via an API system* tweet texts and associated metadata are gathered for one sample of Twitter users resorting to an API system. The collection is guided by keywords, hashtags, or geo-tags to identify which tweets and tweeters are relevant for the study (e.g., Huang and Wong 2016; Storer and Rodriguez 2020).
- *Self-report data collection* data is collected from one sample of Twitter users resorting to inquiry modes, such as online surveys, or in-depth interviews (e.g., Visser et al. 2014; Álvarez-Bornstein and Montesi 2019).

#### Single mode, different samples

This kind of design uses the same mode of data collection for different samples or subgroups. Studies using this approach involve international and regional comparison. Data are collected either using an API system or a self-report mode (e.g., Hayat et al. 2016; Vaccari et al. 2016).

#### Mixed-mode

In the reviewed papers, mixed-mode designs were found in 22 studies. In all these studies, the mixed-mode design was implemented using a sequential approach. More than half of these studies combine a self-report mode − mostly surveys to gather data on Twitter users’ subjective perceptions and opinions − in a first stage of data collection, with data collection via an API system in a subsequent stage. The motivation for mixed-mode designs is either to avoid biases inherent in self-reported data − the researcher gets objective data by collecting data via an API system − , or to allow richer data sets − social media data may not be enough for the study purpose (Salganik 2018, p. 118). These designs produce two matched databases. Specifically, the following strategies (for simplicity are named as A, B and C) were found:

##### Strategy A: self-report data followed by API search

This strategy requires the researcher to conduct a survey on a sample of people that have a Twitter account and then request all survey participants to provide their Twitter ID. Upon acceptance of the participants, the researcher will retrieve their Twitter contents resorting to an API. This strategy can be resumed in the following stages:

- *First stage* Twitter users provide self-reported data by means of a survey questionnaire or in-depth interview.
- *Second stage* the members of the sample volunteer their usernames or IDs for researchers to access their Twitter account and retrieve data and/or metadata via an API system (e.g., Tominaga et al. 2018; Abney et al. 2019).

##### Strategy B: API search followed by self-report data

This strategy starts with a search of the Twitterverse based on keywords, hashtags, or other search term related to the topic under investigation. Twitter users whose data is retrieved are then contacted via the Twitter platform soliciting them to participate in a survey (e.g., sending a message with the invitation and the link to the survey questionnaire). This strategy is common in projects that need to link respondents to their respective auxiliary information (e.g., gender, age, income, education). This kind of information is usually insufficient or is not reliable in big data sources (Daas et al. 2016) making it necessary to implement this kind of data collection strategy. This strategy can be resumed in the following stages:

- *First stage* Twitter data and/or metadata is collected for Twitter users via an API system.
- *Second stage* the same sample of Twitter users provide self-reported data by means of a survey questionnaire or in-depth interview (e.g., D’Heer and Verdegem 2015; Majmundar et al. 2019).

##### Strategy C: API search followed by secondary data

This strategy is adopted in studies in which the geographic location of Twitter users when posting contents needs to be taken in consideration in the analysis. Secondary sources provide supplementary information about the geographical areas under research. This strategy can be resumed in the following stages:

- *First stage* Twitter data and/or metadata is collected for Twitter users via an API system.
- *Second stage* secondary data is collected, mostly from Census or Official statistics, at a spatial or regional (aggregated) level (e.g., Liang et al. 2017; Osorio-Arjona and García-Palomares 2019).

**Fig. 8:**
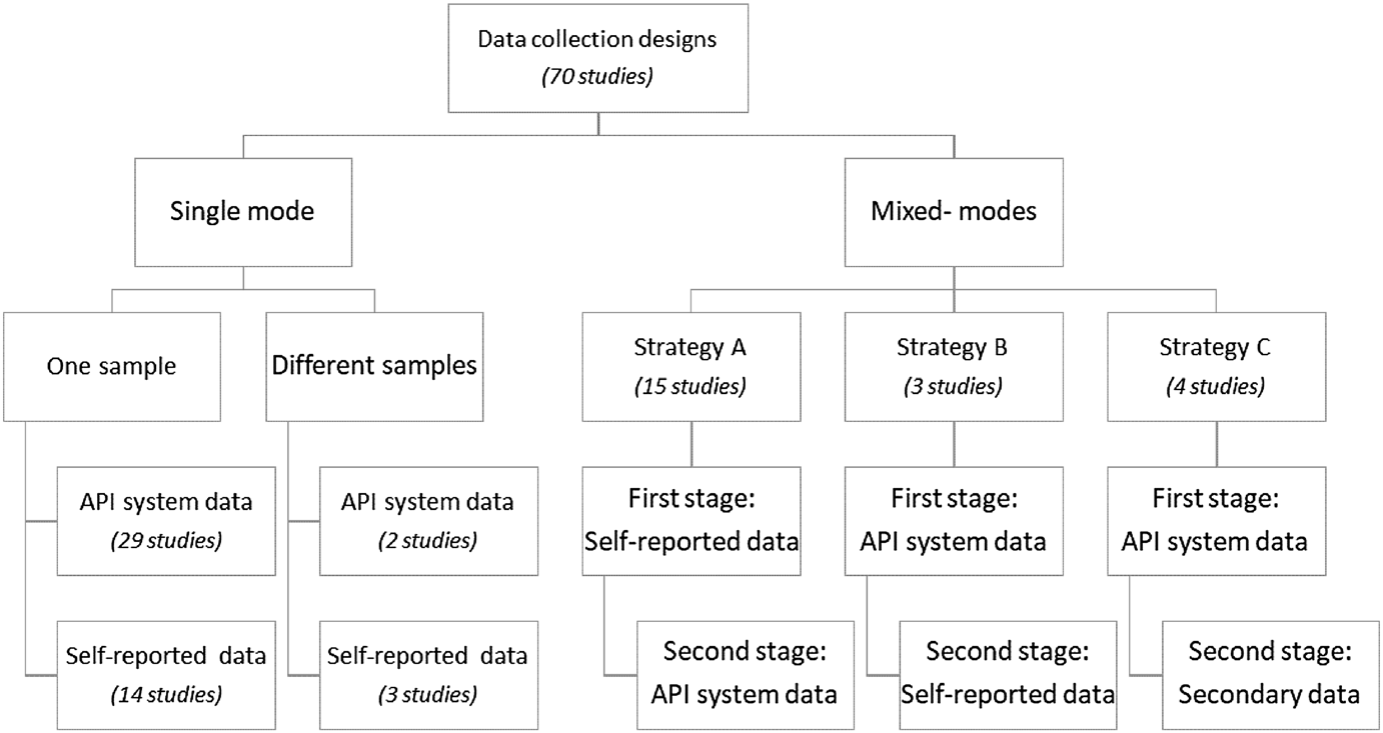
Data collection designs and respective number of studies

Figure 8 summarizes the designs of data collection in the studies covered in the reviewed papers.

In short, 48 studies (69%) relied on a single mode of data collection: 29 studies resorted to an API system applied to a unique sample of Twitter users while 14 studies (19%) resorted to a self-reported mode. Five studies involved more than one sample of Twitter users, but the mode of data collection was unique in each study. A non-ignorable number of studies (18 studies, 26%) combined self-reported data with data gathered via an API system collected for the same sample of Twitter users. Surveys, the most widely used method of data collection in social science research, appears in 50% of the studies either exclusively as the single mode of data collection or in combination with data gathering via API systems.

### Sample size

This subsection addresses RQ5: What is the size of the samples? Regarding sample size in terms of number of Twitter users in the reviewed studies, it was found that: 16 studies (22%) used fewer than 200 Twitter users, while 36 studies (49%) used between 200 and 2000 users. Seventeen studies (23%) used more than 2000 users (3 studies reported more than 1 million participants). Four studies did not report the Twitter user sample size.

The size of the sample is closely connected to the data collection strategy. Table 6 presents statistics summarizing the size of the sample of Twitter users splitting the studies into two categories: (i) “API system data” studies—includes studies in which the data collection design relies exclusively on API systems or involve gathering data via an API system in the first stage of a mixed-mode design;

**Table 6:** Descriptive statistics of Twitter user sample size by data collection strategy

Statistic	“API system data” studies (n = 34)	“Self-report data” studies (n = 32)
Average	297,893	557
Std. deviation	846,179.4	503.5
Minimum	4	12
1st quartile	346	181
Median	2234	330
3rd quartile	36,648	956
Maximum	3,800,000	1,496

ii) “Self-reported data” studies—includes studies in which the data collection strategy relies exclusively on self-reported modes or involve self-reported data gathering in the first stage of a mixed-mode design.

The sample sizes of the “self-report data” studies tend to be lower (average = 557 users, median = 330 users) than the sizes of the “API system data” studies (average = 297,893 users, median = 2,234 users). The distribution of the sample sizes of the “API system data” studies is strongly skewed and highly dispersed which can be explained by the 6 outlier studies that have sample sizes of ≥ 340,000. Excluding these studies from the calculations, the average sample size of the remaining studies drops to 7,380 users (Std deviation = 16,505.8).

### Research validity

This section addresses RQ6: How is research validity addressed? The discussion on the limitations of the study due to methodological shortcomings is only present in 49% of the reviewed papers. As mentioned in §2.4, the Twitter population is likely to suffer from coverage error since not all people use Twitter. Although all the reviewed studies identify their target or study population as Twitter users (either with or without boundaries) (§ 4.4), some warn of the non-generalizability of the findings to other populations and acknowledge that Twitter users may differ either from other social network users (11 studies) or from other general or specific off-line populations (12 studies). Explanations for these issues are provided mostly in the respective Discussion section.

The generalizability of the findings may also be compromised by a non-representative sampling frame, a sampling method that does not guarantee sample representativeness, a sample size inadequate to the study’s objectives, and self-selection bias caused by the pattern of Twitter usage. Details about how the reviewed studies acknowledge these issues are presented as follows.

#### Twitter users differ from other network users

Table 7 presents the citations of the explanations given in the 9 studies that highlight the limitation that the outcomes cannot be generalizable beyond the Twitter network because Twitter users may differ from users of other platforms. The characteristics of Twitter users and the specificities of the Twitter platform are likely explanations for this issue in 4 studies (Gómez-Zará and Diakopoulos 2020; Liang and Fu 2019; Liang and Shen 2018; Tominaga et al. 2018). Three studies postpone investigating whether the findings are valid in other social media platforms for future research (Schaarschmidt and Könsgen 2020; Tominaga et al. 2018; Vaccari et al. 2015).

**Table 7:** Papers and citations of the impact of differences between Twitter users and other social media users

Paper identification	Citation
Amin et al. (2019)	(…) given our work requires the users to have been active both on Twitter and Foursquare, the findings can only be generalized to the population of users who have joined both platforms.
Fiesler and Proferes (2018)	These findings with respect to Twitter may or may not be generalisable for other platforms or contexts [such as] Reddit, Tumblr, Instagram or Facebook.
Gómez-Zará and Diakopoulos (2020)	(…) the Twitter demographic may influence our results. (…) Our results may have been different considering other social media platforms and audiences’ demographics.
Liang and Fu (2019)	Twitter is a social media platform focusing on information sharing. However, there are platforms emphasizing social networking functions, like Facebook.
Liang and Shen (2018)	Twitter could have very different features from other types of social media platforms, such as Facebook and Instagram.
Majmundar et al. (2019)	(…) these findings might not generalize to people with very high social media use, and/or to users of other social media platforms.
Schaarschmidt and Könsgen (2020)	(…) we tested our hypotheses for Twitter accounts only. Further research should scrutinize the observed results for other social media platforms.
Tominaga et al. (2018)	(…) there is still a possibility that characteristics of people depend on the platforms; therefore, we need to verify this issue in the future work.
Vaccari et al. (2015)	(…) Twitter is only one of several such platforms, it is crucial to investigate whether our conclusions can be generalized to broader populations than the one featured in this study.

#### Twitter users differ from off-line or other specific populations

Fourteen studies highlight the limitation that the outcomes are not generalizable beyond the Twitter network because Twitter users may differ from off-line or other specific populations. Table 8 presents the citations of the explanations for this shortcoming.

**Table 8:** Papers and citations of the impact of differences between Twitter users and off-line or other specific populations

Paper identification	Citation
Hswen et al. (2017)	(…) these findings may not generalize to Twitter users with private accounts or to people who do not use Twitter.
Hswen et al. (2018a)	(…) these findings may not generalize to Twitter users with private accounts or to people who do not use Twitter.
Hswen et al. (2018b)	(…) these Twitter users likely differ from individuals with schizophrenia who do not disclose their illness online or who do not use social media. (…) our findings likely do not generalize to individuals who do not use social media.
Jünger and Fähnrich (2020)	(…) limits the generalization of findings because the users of social media sites do not necessarily represent the whole group of interest.
Lee et al. (2020)	Our results cannot be generalized to the broader group of stay-at-home parents who do not participate in this form of social media.
Majmundar et al. (2019)	This sample comprises Twitter users with public profiles limiting generalizability to those with private accounts.
Mihunov et al. (2020)	(…) the results derived from this survey cannot be used to generalize to the general population including, for example non-Twitter users who requested for rescue or Twitter users who did not request for rescue using Twitter.
Mohammed and Ferraris (2021)	As the data were gathered only from students [with Twitter account], the results cannot be generalised to other populations.
Montgomery et al. (2018)	(…) findings from this study are not generalizable to other racial/ethnic groups.
Schaarschmidt and Könsgen (2020)	(…) we tested our hypotheses for Twitter accounts only. Further research should scrutinize the observed results for other social media platforms, such as Facebook, Xing, LinkedIn or Glassdoor.
Storer and Rodriguez (2020)	(…) this sample of Twitter users is not representative of the general population.
Storer et al. (2018)	(…) there are likely significant differences between Twitter users and the general population.
Thomas et al. (2019)	(…) the use of social media data, specifically Twitter, limits generalizability to the broader population.
Vaccari et al. (2015)	(…) we studied a specific population of individuals who posted at least one election-related message, and we cannot determine whether our findings can be generalized to other populations.

#### Sampling frame lacks representativeness

Only 3 papers acknowledge that a sampling frame lacking representativeness may affect the study quality. Baik et al. (2021) mention that “*(…) without a full roster of all Twitter users (…) within a certain period, it is impossible to sample a random sample of Twitter users, which would have been the gold standard (…).”* The other two papers recognise the limitations of resorting to crowdsourcing platforms: Schaarschmidt and Könsgen (2020) refer that “(…) *this study included only U.S. employees recruited *via* a crowdsourcing platform*.” and Tominaga et al. (2018) recognise that “*(…) our study cannot address the issue because we gathered our subjects by crowdsourcing services in which people under 18-year-old cannot use the system.”*

#### Sampling method yields biased samples

Nineteen studies recognise that the sampling method is far from yielding a representative sample of the target population. In 7 studies researchers acknowledge that the sampling methods restrict or compromise the possibility of generalizing the outcomes beyond the scope of the study (Chen 2011; Fischer and Reuber 2011; Hswen et al. 2018b; Majmundar et al. 2018, 2019; Sasaki et al. 2015; Watson 2016). Two studies suggest that random sampling would be the gold standard of sampling in their research (Akyuz et al. 2021; Sashittal and Jassawalla 2019). Table 9 presents citations that illustrate the explanations found about this issue.

**Table 9:** Papers and citations concerning sampling methods deficiencies

Paper identification	Citation
Akyuz et al. (2021)	A convenience sample allows anyone who wants to participate in the study to do so. A random sample would be expected to better represent the target population because neither the researcher nor the participants decide who will participate in the study.
Baik et al. (2021)	(…) one clear bias in using this [nonrandom selection] method is that any user that tweets more frequently will be more likely to be selected.
Cavazos-Regh et al. (2017)	(…) the “typical Twitter user” (…) is not an exact match of our sample of Tweeters.
Chen (2011)	(…) a nonprobability sample was obtained using convenience snowball sampling, (…) this sampling method does not offer generalizability of results (…).
Fischer and Reuber (2011)	(…) the sample [of entrepreneurs] we chose to use included only business-to-business firms, and no business-to-consumer firms. (…) This sample limitation does mean, however, that the theoretical insights should not automatically be assumed to generalize to all contexts.
Hswen et al. (2017)	(…) given that we generated a convenience sample of Twitter users who self-identified as having schizophrenia through manually searching the Twitter platform, these individuals also may not be representative of the broader group of Twitter users who self-identify as having a schizophrenia spectrum disorder.”
Hswen et al. (2018b)	(…) we employed a convenience sampling approach to generate the group of Twitter users who self-identify as having schizophrenia This sampling method further limits generalizability of these findings.
Kobayashi et al. (2019)	(…) the respondents in the present study were not randomly sampled from the population (i.e., they were Japanese Twitter users who follow at least one media account and at least one member of the Japanese Diet), so we cannot preclude potential systematic sampling bias.
Lee et al. (2020)	(…) snowball sampling can be prone to bias because of the correlations between individuals. That is, Twitter users in the subsequent round of data collection are not independent of Twitter users in the first round of data collection, which could ultimately yield biased or inconsistent results. For these reasons, results of the study should be interpreted with caution.
Majmundar et al. (2018)	This sample comprises Twitter users with public profiles limiting generalizability to those with private accounts.
Majmundar et al. (2019)	(…) the study sample is non-representative of U.S. population, which limits generalizability of the findings.
Montgomery et al. (2018)	(…) the use of a convenience sample limits the ability to generalize the findings to the national population.
Pentina et al. (2013)	The method of snowball sampling could have introduced selection bias in the data collection.
Sasaki et al. (2015)	(…) our sample was limited to users in only one country (…) Therefore, we recommend caution in generalizing the results to users in other countries.
Sashittal and Jassawalla (2019)	Random samples of all Twitter users are left to future research.
Storer et al. (2018)	Twitter users are not a representative sample. (…) This sample is generalizable only to those who respond to and follow social issues on Twitter.
Visser et al. (2014)	This survey was originally disseminated via tweets from the authors’ Twitter accounts, and our tweets were then retweeted many times by many people, including some influential educators. Due to the inability to track how many people received or read these tweets, the researchers were unable to obtain the precise numbers necessary to calculate and provide an exact response rate
Yu et al. (2019)	Because of the large number of scientific tweeters, it is difficult to analyze a statistically representative sample (…) so the results may overrepresent the highly active scientific tweeters.
Watson (2016)	Neither the journalists nor Twitter users in this study represent a scientific random sample (…). Findings should not be generalized beyond the Gulf Coast newspaper journalists and most followed Twitter users in the sample.

#### Insufficient sample size

Only five studies mention that sample size or the achieved response rate were not good enough to grant validity to the findings (Table 10). Two studies postpone replicating the research with bigger samples sizes for future research (Qiu et al. 2012; Shin 2020).

**Table 10:** Papers and citations concerning sample size insufficiencies

Paper identification (Authors)	Citation
Brady (2016)	The sample size for this project was small and posed a limitation to the strength of the results.
Gómez-Zará and Diakopoulos (2020)	Given the low response rate (…)
Majmundar et al. (2018)	(…) sample size of this study also limits findings’ generalizability (…)
Qiu et al. (2012)	While our sample size is comparable to other studies on personality and social media (…) future studies should include more participants in order to verify our findings.
Shin (2020)	(…) the findings were drawn from a relatively small sample of Twitter users (…). Future studies are needed to expand the sample size to more accurately assess the demographics and media behaviors of Twitter users

#### Self-selection bias

A small number of studies (3) acknowledged self-selection bias—caused by the fact that the pattern of Twitter use can negatively impact sample representativeness—as a potential problem for outcome validity. Baik et al. (2021) refer that “*one clear bias in using this method* [API system search] *is that any user that tweets more frequently will be more likely to be selected*.”. Storer et al. (2021) stress that the behavior of Twitter users when in the platform is not uniform: “*individuals use this platform in a variety of different ways (i.e., consuming news, connecting with work colleagues, *etc*.) (…). This sample is generalizable only to those who respond to and follow social issues on Twitter.”* Hswen et al. (2017) mention that the information users chose (or not) to make public restricts the scope of the findings: “*they may differ from individuals with schizophrenia who choose not to disclose their illness online*.”

## Discussion

The general information of the studies revealed that most of the research concentrated in the United States (34 studies) and is conducted in an academic context (68 studies). Most research falls into the domains of Information & Communication (16 studies), Information & Computer Science (11 studies), and Social & Behavioral Sciences (11 studies), which are key areas of social science and computational social science (Cioffi 2010; Investopedia 2021). Much of the research is either descriptive (28 studies) or explanatory (29 papers), which aligns with the pattern of social science investigation—describing the phenomenon under study and identifying its causes and effects are most the common research interests of social scientists (Babbie 2017, p.22). However, four of the reviewed studies acknowledge that the cross-sectional nature of the data did not allow cause-effect relationships to be proved, but merely identified associations between variables; they claimed the research would benefit from adopting either a longitudinal or a before-and-after experimental design (Hofer and Aubert 2013; Majmundar et al. 2019; Thomas et al. 2019; Moshkovitz and Hayat 2021).

This section highlights the factors that represent the challenges in sampling plans for applied social science research in Twitter, namely target populations, sampling frames, sampling methods/strategies, sample size and data collection methods/strategies. Additionally, the impact of these factors on research generalizability is discussed in the light of the literature review.

### Target populations

The target population of Twitter users is in most of the studies bounded resorting to Twitter-specific features that describe what people write on Twitter (“tweet object) (18 studies), what people do or are on Twitter (“user object”) (17 studies), or where people are when they tweet (“geo-object”) (4 studies).

Even though the target population involves, in all studies, Twitter users—a consequence of papers’ inclusion criteria—in 23 studies the respective authors acknowledged that the findings are likely to suffer from coverage error and for that reason could not be generalised outside the Twitterverse. This alert can be seen, in a first moment, as unnecessary since in a study targeted at “Twitter users” it is not expected to extrapolate conclusions beyond that universe. However, the decision to disclosure it as a potential limitation of the study is a sign of social science researchers’ prudence by recognising that Twitter cannot fully replace a study conducted in the “real-world” or even in other platforms.

### Sampling frames

Most studies (46) were found to be conducted without a sampling frame and some others resorted to lists that are imperfect representations of the Twitter population, e.g., online panels (11 studies) or crowdsourcing lists (4 studies). The reason for such a scenario is that there is no public and easily available list of Twitter users, thus forcing researchers to either create their own lists—which is difficult to accomplish for large populations—or use “proxy” lists to approach Twitter users; however, these cases suffer from a serious risk of lack of coverage (Ruths and Pfeffer 2015). Online panels or platforms of crowdsourcing services allow easy access to Twitter users, but these lists are hardly representative of the Twitterverse because these sampling frames are not built with the purpose of matching that population. Although online panels are representative of the general adult population, they cannot be assumed to be representative of the Twitter population as previous investigation suggests that this population is socio-demographically different from the general offline population (Blank 2017; Pew Research Center 2019; Hootsuite 2020). In the few studies in which researchers were able to build a tailored sampling frame (8 studies), the research covered either small or very specific populations—e.g., psychologists who were directors of professional associations (Brady 2016), scientific tweeters (Yu et al. 2019) or educators (Visser et al. 2014). Only 4 studies used a conceptual sampling frame “created” by random generation of Twitter users’ IDs; this is likely to be explained firstly by the fact that not all social scientists possess the demanding computational knowledge required to implement such a procedure, and secondly because while creating a list of Twitter users’ IDs by random number generation is feasible for the “population of all Twitter users”, it is difficult to implement for bounded populations (which was the case of most studies). However, Berzofsky et al. (2018) present promising results of this approach to select probability-based samples on Twitter if the range of ID numbers that correspond to active Twitter users can be identified in advance.

### Sampling methods and strategies

The sampling strategies undertaken when no sampling frame is available are based either on non-random methods (convenience, purposive or snowball sampling) or data search-based. On one hand, this is a consequence of the lack of sampling frames which impedes random selection (Groves et al. 2009, p. 94; Couper 2013); on the other, it is a choice to guarantee an efficient sampling procedure: selecting a sample of Twitter users by first locating tweets they posted containing the topic or event of interest is an efficient strategy to locate hard-to-reach populations or topic/event specific population. The selection is based on the dependent variable which guarantees the users are selected within the scope of the study; however, there is a risk of oversampling the users who are more active on the platform and under sampling those who rarely use or seldom post contents (Bruns and Stieglitz 2013; Zhang et al. 2018).

The randomness of a sample—each element has a nonzero probability of being chosen—is of the utmost importance for the social scientific methodological integrity as a sample selected randomly is regarded as a valid representation of the total population. There is no known method of obtaining a random and representative sample of all Twitter users, so sample selection based on an initial data search using keywords or hashtags was the most common approach namely in studies that were topic- or event- centered. Even though Twitter promises “random” samples of their data when using their API systems, Twitter samples are not free of criticism. Pfeffer et al. (2018) conducted an experiment to test the sampling procedure of Twitter’s API by inducing tweets into the feed in such a way that they appear in the sample with great certainty. The authors found that 100 accounts were enough to manipulate the data stream for a globally important topic. Despite sharing (parts of) its data, Twitter does not reveal details about its sampling mechanisms (Ruiz-Soler 2017), which leaves researchers with no control over sample selection and makes it difficult to design strategies capable of dealing with potential bias. The reviewed papers that based sample selection on data search resorting to Twitter’s APIs (31 studies) are sparce in methodological details about sample selection, which is a likely sign of the lack of information that the researchers themselves have of the sampling process. Researchers must trust Twitter to supply them with methodologically sound samples while dealing with all kinds of other problems, such as bias and ethical issues (Bruns 2019).

Ground theory from survey methodology states that random sampling must be encouraged in applied research. This is also desirable for Twitter context research and can be implemented by random generation of users’ ID numbers. Although few studies used this approach (only 4), it is the most promising for implementing random selection when no list of Twitter users is available. Despite the risk of generating numbers that do not correspond to active users or that are out of the scope of the study—which brings some inefficiency to the process—, it has the advantage of giving all users the same probability of selection and thus avoids samples biased towards the most active and most proficient users. It would be interesting to transpose the Random Digit Dialling of phone numbers—which is also based on randomly generated numbers—to the sampling of social media users as this is a highly regarded method of sampling frame creation that is commonly used by research organizations around the globe. However, this strategy will bring the challenge of detecting out-of-the-scope users such as bots (Alothali et al. 2018) since random number generation is likely to generate a lot of numbers that do not match researchers’ interest.

### Sample size

Samples sizes in the reviewed papers ranged from “small” (up to 2000 users) to “big” but there is a strong skewness towards smaller samples: 70% of the studies used samples of less than 2000 Twitter users; only 4% reported samples sizes over 1 million users. Given that Twitter is a big data source providing access (at least theoretically) to huge datasets, one would expect bigger samples sizes. That was not the case though. Likely explanations for this might lie in the fact that some research was focused on very restricted domains or populations (e.g., Watson 2016; Lee et al. 2020) or had an exploratory purpose where the precision and accuracy requirements are less demanding so there is no need for large sample sizes (e.g., Brady 2016; Baik et al. 2021).

The size of the sample is to a great extent also closely connected to the data collection strategy, namely whether the strategy relies on gathering data via an API system or mostly on self-report data. When splitting the analysis by type of data collection strategy, it is evident that the sample size in studies employing surveys or other self-reported mode (32 studies) tends to be lower (average = 557 users) than in studies that use exclusively (or rely heavily on) API systems (average = 297,893 users) (34 studies). The average sample size of self-reported studies is in line with social science practice since sample size in surveys targeted at specific populations with no stratification is usually dimensioned between 200 and 500 cases (Sudman 1983). However, the size of samples relying on data search is low given the huge dataset sizes that the Twitterverse potentially offers. Despite the impressive scale of Twitterverse data, researchers start looking at large datasets as a means to an end and not as an end in themselves (Callegaro and Yang 2018; Salganik 2018, p. 17).

### Data collection methods

The data collection designs found in the reviewed papers were classified as: (i) Single mode, one sample, (ii) Single mode, different samples, and (iii) Mixed-mode. The most frequent design is “single mode, one sample” in which data is collected from one sample, using a single mode of data collection. In 29 studies, Twitter data and/or metadata is gathered for one sample of Twitter users resorting to an API system; on the other hand, data were collected in 14 studies through the exclusive use of a self-report mode, specifically surveys. In mixed-mode designs, data are collected for one sample of Twitter users, with different modes for different stages of the study. More specifically, 15 studies collected data for a sample of Twitter users by means of a survey questionnaire in a first stage, and the same sample is asked to volunteer their Twitter usernames or IDs in a second stage so that researchers can access their Twitter data and/or metadata via an API system. Despite the huge amount of data that can be retrieved from social media platforms, social media data is not enough in some studies and other data sources must be used to supplement and enrich the data gathered within the Twitter platform. This is demonstrated by the 22 studies that employed mixed-mode designs of data collection. Even though some argue that sampling is “an artefact of a period of information scarcity” (e.g., Mayer-Schönberger and Cukier 2013), the systematic review presents evidence that survey sampling is a central necessity even in times of information abundance.

Regarding data collection, research would benefit from exploring mixed-mode designs instead of focusing solely on Twitter data retrieval. Twitter data may not be enough for the research goals since it is mostly composed of events and usually provides very little or no information on the user that generated the data. Social media studies are frequently limited by not reporting demographic data because of the difficulty in retrieving this information from publicly available online data sources. Twitter suffers from this problem, which is a severe limitation since variables such as age, sex, income, education are crucial in social science research for multivariate analyses or subgroup comparisons. From a social science perspective, Twitter data is case rich (big samples) but variable poor (few covariates), which is why the complementarity of other modes is essential in some studies (Couper 2013; Callegaro and Yang 2018; Salganik 2018, p. 24). This suggests that contrary to what some have predicted (e.g., Savage and Burrows 2007), Twitter will not replace surveys in social science research; however, it opens new avenues of investigation that will enrich the understanding of social issues if both approaches work together instead of separately. Surveys are the best mode to collect data at an individual level and are the most used tool to supplement Twitter data in social science research. Researchers should give further attention to refining the design to articulate data from these two different sources (Callegaro and Yang 2018).

### Research validity

Less than half the studies (36 studies) acknowledge that factors related to the design and implementation of the sampling plan may impact the outcomes and compromise findings’ generalizability. Twenty-three studies highlight coverage error as a major problem when conducting applied social science research using Twitter. This concern is justified by the limited penetration of the Twitter platform—nearly 400 million active users worldwide—much behind Facebook, YouTube, or WhatsApp which each have more than 2000 million active users worldwide (Statista 2022b). With such a low use, it is hard to accept that the Twitter population is a reliable representation of the offline population or of the population using other platforms.

Sample selection is noted as a major problem in 19 studies, but no details are provided about the magnitude of the impact on the findings of using specific sampling methods. The development and implementation of strategies to deal with these issues, such as adopting random sampling instead of non-random sampling (e.g., Pentina et al. 2013; Kobayashi et al. 2019; Akyuz et al. 2021) or sample weighting (Kobayashi et al. 2019), are postponed to future research.

Self-selection bias is acknowledged in three papers. The usage pattern of social media in general and of the Twitter platform in particular is highly diverse across users, with a few contributing with many tweets and the remaining posting hardly anything (Bruns and Stieglitz 2013; Nielsen 2017; Pew Research Center 2019). This impacts sample selection since the most active users are more likely to be selected—namely in a sampling strategy based on data search—and there is no guarantee that they are representative of the Twitterverse. This problem must be addressed not only by designing sampling strategies to reduce its occurrence but also by developing weighting schemes to attenuate its effect. Just as in surveys, paradata (i.e., data about the process of answering the survey itself, collected by systems and third parties, before, during, and after the administration of a questionnaire) are used to build weights to correct for nonresponse bias (Nicolaas 2011; Olson 2013) in social media, metadata, such as number of posts and number of followers, can be used to build weights aimed to account for different likelihoods of the selection of Twitter users. The future of Twitter-based research must look for answers to these issues so that a solid body of knowledge is acquired, thus contributing to reliable social science and computational social science research (Ruths and Pfeffer 2014).

## Conclusion

This paper presents a systematic quantitative literature review of the design and implementation of sampling plans to select samples of Twitter users in applied social science studies. Seventy-three papers were identified as reporting evidence of the methodology adopted to select samples of Twitter users, and were analysed to find empirical answers to questions such as which sampling frames are best to represent Twitter users, which sampling strategies should be employed to select representative samples of Twitter users and which strategies of data collection should be adopted.

Results show that applied social science studies are conducted either without a sampling frame or with a sampling frame that does not adequately represent the population of Twitter users. Sampling strategies are to a great extent conditioned by this fact. Twitter users are selected either resorting to non-random methods—mostly convenience or snowball sampling—or by searching the tweets they posted containing specific keywords or hashtags. Regarding data collection designs, most of the studies relied on a single mode of data collection—namely, via an API system—applied to a unique sample of Twitter users. A non-ignorable number of studies combined self-reported data, namely surveys, with data gathered via an API system collected for the same sample of Twitter users. Surveys, the most widely used method of data collection in social science research, appears in 50% of the studies either exclusively as the single mode of data collection or in combination with data gathering via API systems. This is a significant sign that many research questions cannot be fully answered exclusively through social media data and require mixed-mode approaches to obtain a deep understanding of the phenomenon.

As in any other literature review, this work is limited by the search terms used and the databases searched (Pickering and Byrne 2014). To try to minimize this limitation, similar and commonly interchanged terms were included as well as a search on databases focused on social sciences and related fields. However, there may still be some relevant works that were not captured by the search criteria. Despite this fact, the collected evidence suggests that sampling considerations should become central components of a project’s research design, and the strengths and limitations of different sampling plans should be explicitly included in the discussion of results. Based on the review undertaken, it is evident that we are still far from that scenario since less than half the studies discussed the limitations and the impact on research validity caused by using Twitter.

The research conducted on Twitter is vast and has been applied to a variety of domains, including social sciences. However, the use of this platform raises new quality issues that must be properly addressed because only when the limitations and challenges inherent to a data source are acknowledged, can a phenomenon be understood and the research findings validly interpreted (Callegaro and Yang 2018).
